# Tauopathy and Movement Disorders—Unveiling the Chameleons and Mimics

**DOI:** 10.3389/fneur.2020.599384

**Published:** 2020-11-05

**Authors:** Jacky Ganguly, Mandar Jog

**Affiliations:** Movement Disorder Centre, London Health Sciences Centre, University of Western Ontario, London, ON, Canada

**Keywords:** tauopathy, movement disorders, chameleons, mimics, MAPT

## Abstract

The spectrum of tauopathy encompasses heterogenous group of neurodegenerative disorders characterized by neural or glial deposition of pathological protein tau. Clinically they can present as cognitive syndromes, movement disorders, motor neuron disease, or mixed. The heterogeneity in clinical presentation, genetic background, and underlying pathology make it difficult to classify and clinically approach tauopathy. In the literature, tauopathies are thus mostly highlighted from pathological perspective. From clinical standpoint, cognitive syndromes are often been focussed while reviewing tauopathies. However, the spectrum of tauopathy has also evolved significantly in the domain of movement disorders and has transgressed beyond the domain of primary tauopathies. Secondary tauopathies from neuroinflammation or autoimmune insults and some other “novel” tauopathies are increasingly being reported in the current literature, while some of them are geographically isolated. Because of the overlapping clinical phenotypes, it often becomes difficult for the clinician to diagnose them clinically and have to wait for the pathological confirmation by autopsy. However, each of these tauopathies has some clinical and radiological signatures those can help in clinical diagnosis and targeted genetic testing. In this review, we have exposed the heterogeneity of tauopathy from a movement disorder perspective and have provided a clinical approach to diagnose them ante mortem before confirmatory autopsy. Additionally, phenotypic variability of these disorders (chameleons) and the look-alikes (mimics) have been discussed with potential clinical pointers for each of them. The review provides a framework within which new and as yet undiscovered entities can be classified in the future.

## Introduction

Tauopathies are a heterogeneous group of neurodegenerative disorders, pathologically characterized by neuronal and/or glial inclusions of the microtubule-binding protein, tau. Heterogeneity spans many domains from the clinical presentation, anatomical localization, genetic variations, and radiological and pathological signs. Neuroanatomical vulnerability may be a key to the heterogeneity (the concept of “molecular nexopathies”) (1). Many factors can be implicated including “strain” specificity of the tau protein, biochemical property of the abnormal protein according to its post-translational modification, “prion-like” propagation capacity, interaction with other co-existent proteins like alpha-synuclein or TDP43, seeding or “permissive templating” property, intrinsic vulnerability of the affected structure, genetic, and epigenetic factors and environmental influences (1, 2).

The spectrum of tauopathy is still unfolding and transcending beyond the domain of primary tauopathies. While secondary tauopathy from autoimmune insult like in anti-IgLON5 disease brings up the topic of complex interaction between autoimmunity and neurodegeneration (3), geographically isolated tauopathies highlight the environmental impact. Apart from this, some novel tauopathies are also increasingly being described in the literature (4).

Clinically, tauopathies present as movement disorders, dementia, and motor neuron disease, either in isolation or in varied combinations (5), based on the vulnerable anatomical structures being affected by the pathological protein accumulation. In terms of genetics, MAPT gene containing N terminal domain (N1, N2) and microtubule binding domain (R1, R2, R3, R4), on chromosome 17q21 encodes the protein tau. Due to alternative splicing of the MAPT gene, three repeat (2N3R, 1N3R, 0N3R) or four repeat (2N4R, 1N4R, 0N4R) tau isoforms are formed (6). On the other hand, depending upon numerous single nucleotide polymorphisms (SNPs) and a 900kb inversion, H2 and H1 haplotypes of MAPT gene are formed and have impact on the phenotypic presentation (7).

In the literature, tauopathy has been discussed mostly as a pathological entity with its detailed pathological intricacies. Pathological confirmation of the diagnosis of tauopathy mostly depends on autopsy findings. However, pathological diagnosis is often confounded by the presence of multiple other proteins and thus it becomes difficult to determine whether the accumulated tau is pathological or an innocent bystander. *In vivo* biomarkers like CSF tau and tau-PET imaging are still research-based tools. Additionally, each of these tauopathies has some clinical and radiological signature that can predict the underlying genetics and pathology.

In order to clarify this complexity of heterogeneity of tauopathies, in this review, we have approached tauopathy from a clinical standpoint highlighting mainly the movement disorder perspective, focused on the clinical presentations (chameleons) and their phenotypic look-alikes (mimics). A critical review of the current status of the classification of tauopathies will be followed by the clinical spectrum of primary, secondary, and geographically isolated tauopathies to understand the heterogeneity. Potential clinical and radiological clues will be discussed for each of them. Finally, a practical approach is presented to guide the clinician in day to day practice. Specifically, the phenotype of familial frontotemporal dementia with parkinsonism has been clinically dissected further at the end, because it is one of the commonest overlapping phenotypes of tauopathies.

## Classification of Tauopathies—Current Status and Pitfalls

**Figure 1:**
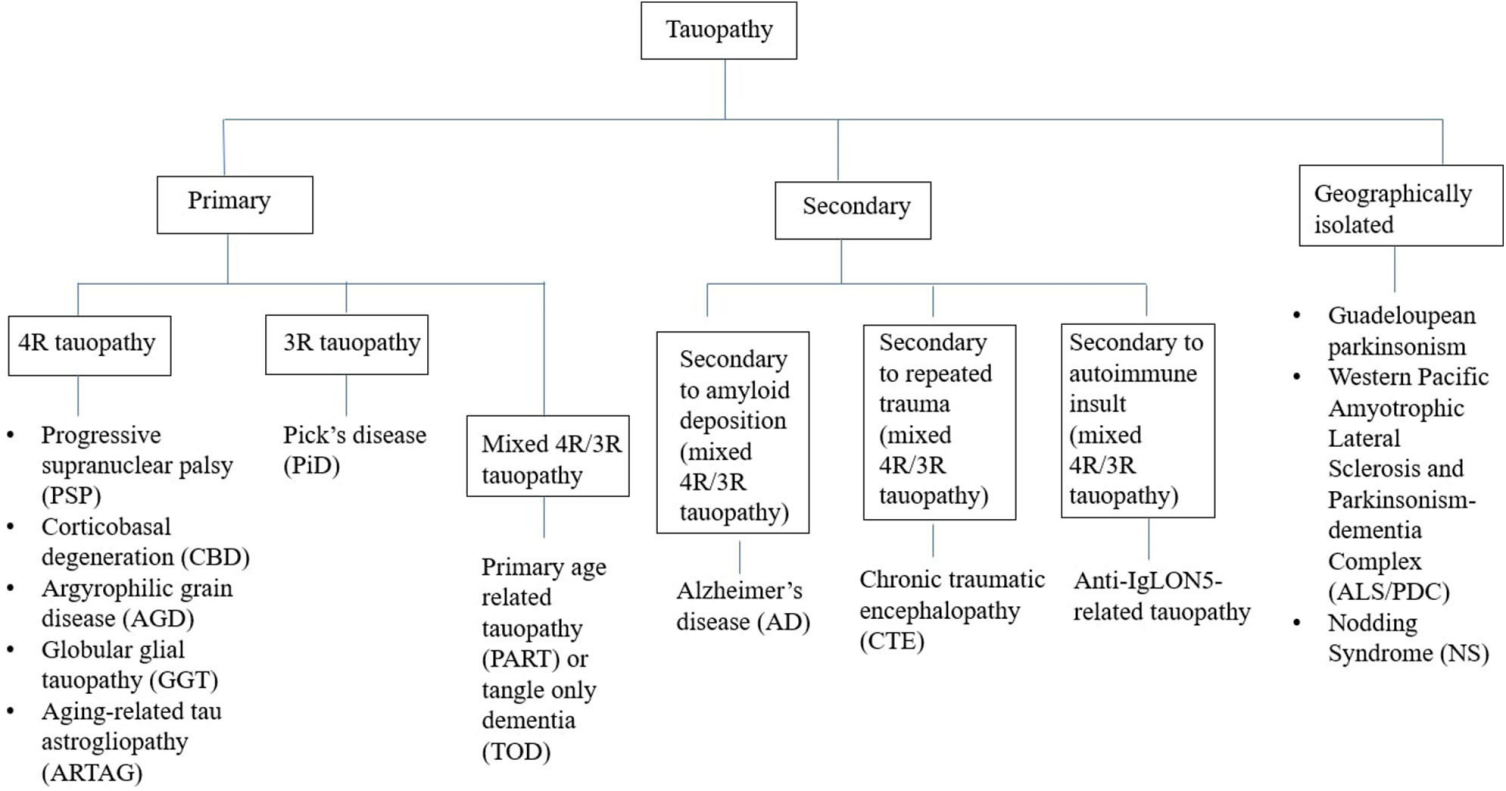
Classification of tauopathies.

Tauopathies have been conventionally classified from a pathological perspective into two groups—(A) Primary tauopathies where tau is the predominant pathology including three repeat (3R-) and four repeat (4R-) tauopathies, (B) Secondary tauopathies where additional etiologies (e.g., amyloid, trauma, and autoimmune) are involved for tau deposition (Figure 1) (8). However, some tauopathies are geographically isolated like Guadeloupean parkinsonism (9), Western pacific amyotrophic lateral sclerosis and parkinsonism-dementia complex (ALS/PDC) (10), and Nodding syndrome of northern Uganda (11). While exact etiopathogenesis of these geographically isolated tauopathies are still unknown, environmental impact (discussed later) has been highlighted in many studies. Thus, whether to include them in the group of secondary tauopathies or not, is still a matter of debate.

Recently, Hõglinger et al. (12) have highlighted syndromic classification of tauopathy based on the predominant domain affected (cognitive or motor):

1. Cognitive syndromes: Behavioral variant of frontotemporal dementia (bvFTD), non-fluent agrammatic variant of primary progressive aphasia (nfavPPA), semantic variant of primary-progressive aphasia (svPPA), and amnestic syndrome of hippocampal type (AS).
2. Motor syndromes: Richardson syndrome (RS), Parkinson syndrome (P), corticobasal syndrome (CBS), primary gait freezing (PGF), cerebellar syndrome (C), and primary lateral sclerosis (PLS).

However, a primary tauopathy like progressive supranuclear palsy (PSP) or corticobasal degeneration (CBD) can present with different cognitive and motor syndromes (chameleons) and many a times there is phenotypic overlap of cognitive and motor syndrome like in familial FTD with parkinsonism linked to MAPT (FTDP-17).

Apart from these, tauopathies can be classified based on the etiology like genetic (e.g., MAPT related), autoimmune (e.g., anti IgLON5 related), traumatic (e.g., chronic traumatic encephalopathy), etc. It can also be classified based on the area of brain predominantly involved like frontal cortex (e.g., behavioral variant frontotemporal dementia/bvFTD, progressive supranuclear palsy-frontal variant/PSP-F), parietal cortex (e.g., corticobasal syndrome/CBS), peri-sylvian (e.g., progressive nonfluent aphasia/PNFA), limbic (e.g., argyrophilic grain disease/AGD), brainstem (e.g., progressive supranuclear palsy-Richardson's type/PSP-RS, anti IgLON5 related), or cerebellum (e.g., PSP-C).

### Primary 4R- and 3R-Tauopathies

In this large group of primary tauopathies, CBD, GGT, AGD, and PiD are primarily pathological diagnosis where as corticobasal syndrome (CBS) is a clinical term. PSP can be described as both, a pathological or a clinical entity. In this review, PSP and CBS have been discussed with their phenotypic presentations (chameleons) and look-alikes (mimics). PNFA/PPA-G has also been discussed because it is primarily a clinical diagnosis and its pathology is mostly FTLD-tau. In the literature, GGT, AGD, and PiD have been traditionally discussed from a pathological standpoint. We have highlighted the clinical and radiological clues for suspecting GGT, AGD, and PiD clinically before confirmatory autopsy. We have sub-classified primary tauopathies according to the predominant clinical presentation like, movement disorder (PSP, CBS), language dysfunction (PNFA) and cognitive dysfunction or mixed (GGT, AGD, and PiD). However, movement disorders can be associated with the second and the third subtype in varied proportions.

### Predominant Movement Disorder Presentation

#### Progressive Supranuclear Palsy (PSP)

Axial rigidity, facial dystonia, retrocollis, vertical supranuclear gaze palsy (VSGP), early postural instability, and pseudobulbar palsy are clinical pointers for classic PSP or Richardson's phenotype (PSP-RS). Apart from this, PSP can have varied phenotypic presentations (chameleons) like parkinsonian type (PSP-P), progressive gait freezing (PSP-PGF), etc. (Table 1) (13–15). According to the latest MDS criteria, four core clinical features should be assessed for varying levels of certainty for PSP pathology: (i) oculomotor dysfunction (e.g., VSGP, slow vertical saccades, square wave jerks, and eyelid apraxia), (ii) postural instability within 3 years (e.g., spontaneous loss of balance, unprovoked falls, tendency to fall on pull-test), (iii) akinesia (e.g., progressive gait freezing, akinetic rigid, predominantly axial parkinsonism), and (iv) cognitive dysfunction (e.g., non-fluent aphasia, apraxia of speech, frontal cognitive/behavioral presentation) (16). Levodopa resistance (<30% improvement of the MDS UPDRS III score on a levodopa challenge), dysphagia, hypokinetic spastic dysarthria and photophobia are useful suggestive features of PSP (16). Dorsal midbrain atrophy is the characteristic radiological finding leading to “Morning glory,” “Mickey Mouse” signs on axial MR images and “Hummingbird,” “Penguin silhouette” signs on sagittal MR images (17). Midbrain/Pons (M/P) ratio < 0.52, midbrain AP diameter measurement < 9.35 mm (18), MR Parkinsonism Index (MRPI) > 13.55 (19), and MRPI 2.0 > 2.18 for PSP-P, > 2.50 for PSP-RS (20) are other helpful radiological signs. MAPT H1 haplotype, specially H1c sub-haplotype and recently described H1d, H1g, and H1o sub-haplotypes of MAPT are associated with increased risk of PSP. Classic PSP pathology is characterized by “tufted astrocytes” and “globose” neurofibrillary tangles. Predominantly PSP pathology is seen in the progressive gait freezing phenotype (PSP-PGF) and in Richardson's phenotype (PSP-RS), whereas in the other variants of PSP, the pathology is often mixed or of non-PSP pathology (21). Alzheimer's disease (AD) pathology and argyrophilic grains (AG) are commonly associated co-pathologies (22). Overall, the phenotypic presentation of PSP depends on the brain area that is more vulnerable to the pathological protein accumulation like frontal lobe in PSP-F, parietal lobe in PSP-CBS, temporal lobe in PSP-SL, midbrain in PSP-RS, basal ganglia (post-synaptic striatal) in PSP-P, pons in PSP-PGF and cerebellum in PSP-C (Table 1) (23, 24).

**Table 1:** Chameleons of PSP and CBS.

**Disease entity**	**Phenotypic presentations (Chameleons)**	**Clinical clues**
Progressive supranuclear palsy (PSP) (13, 16, 25)	PSP-Richardson's syndrome (PSP-RS)	Vertical supranuclear gaze palsy (VSGP), slowing of vertical saccades, early postural instability within 3 years, axial rigidity, retrocollis, hyperactivity of frontalis and procerus muscle (“Reptilian stare,” “Procerus sign”)
	PSP-parkinsonism (PSP-P)	Initially mimics Parkinson's disease (PD), prominent axial symptoms, attenuated response to levodopa, along with VSGP or slow vertical saccade, hypokinesia without decrement, micrographia without decrement in script size (26), freezing of swallowing (27) are helpful clinical clues
	PSP-Progressive gait freezing (PSP-PGF)	Gait ignition failure, start hesitation, progressive freezing of gait (FOG) within 3 years, along with VSGP or slow vertical saccade, stuttering or stammering speech, axial rigidity without appendicular rigidity, fast micrographia, rapid hypophonia, or tachyphemia
	PSP-Corticobasal syndrome (PSP-CBS)	VSGP/slow vertical saccade with features of CBS like delayed initiation of horizontal saccade, limb apraxia, dystonia, myoclonus, cortical sensory loss
	PSP-speech/language disorder (PSP-SL) or PSP-PNFA	VSGP or slow vertical saccade with features of PNFA like progressive apraxia of speech (AOS), agrammatism, phonemic errors
	PSP-frontal variant (PSP-F)	VSGP/slow vertical saccade with frontal cognitive/behavioral presentation like apathy, dysexecutive syndrome, reduced phonemic verbal fluency, impulsivity, disinhibition, perseveration
	PSP-postural instability (PSP-PI)	Isolated postural instability within 3 years (repeated unprovoked falls or fall during pull test)
	PSP-ocular motor (PSP-OM)	Isolated VSGP/slow vertical saccade/macro square wave jerk or eyelid opening apraxia
	PSP-primary lateral sclerosis (PSP-PLS) (28)	PSP phenotype with marked upper motor neuron (UMN) signs (may be a clinical clue for underlying GGT pathology)
	PSP-cerebellar ataxia (PSP-C) (29)	Progressive truncal and limb ataxia
		Can mimic multisystem atrophy (MSA-C) or idiopathic late onset cerebellar ataxia (ILOA); early falls, VSGP, no dysautonomia, cognitive dysfunction are helpful clinical clues for PSP-C
Corticobasal syndrome (CBS)	Classic CBS phenotype (CBD-CBS) (30)	Asymmetric parkinsonism, limb dystonia, myoclonus, saccadic apraxia, ideomotor apraxia, cortical sensory deficits, alien limb phenomena
	Non fluent/agrammatic variant Primary progressive aphasia phenotype (CBD-PNFA) (30)	Apraxia of speech (AOS), agrammatism
	Frontal behavioral-spatial syndrome (FBS/ CBD-bvFTD) (30)	Executive dysfunction, disinhibited behavior, personality changes
		Mimics bvFTD, but with additional visuospatial and visuoconstructive deficits
	PSP-RS-like phenotype (PSPS/CBD-RS/CBD-PSP) (30)	VSGP or slowing of vertical saccade, axial rigidity, postural instability, early falls
	Amnestic phenotype (31)	Mimics AD like dementia at onset, additional asymmetric motor/sensory signs, hyperreflexia, gait impairment, parkinsonism, dystonia are clinical clues
	Posterior variants clinically presenting with Posterior cortical atrophy (CBD-PCA), Gerstmann-variant or Balint syndrome (32)	Symmetric bi-parietal syndromic presentation with asymmetric progression, progressive visuospatial impairments, fluent aphasia, posterior alien hand, apraxia, agraphia, acalculia, optic ataxia, oculomotor apraxia, simultagnosia with parkinsonism, myoclonus
	Progressive dysarthria and orofacial apraxia variant (33)	Presents with progressive loss of speech output, orofacial apraxia (OFA) for lower facial and tongue movements, later development of myoclonus, limb apraxia, akinetic-rigid parkinsonism
	Prominent pseudobulbar effect and dysarthria, emotional lability variant (34)	Presents with spastic dysarthria, pathological laughter/crying, later development of asymmetric rigidity, dystonic posturing
	Progressive conduction aphasia (35)	Presents with progressive language problem with preserved fluency and comprehension but with paraphasia and marked impairment in repetitions of words or phrases
	Frontal-type gait impairment (36)	Presents with difficulty to initiate gait, imbalance during walking, marked anxiety for falling, upper limb dyspraxia, paratonia, frontal release signs

#### Corticobasal Syndrome (CBS)

Asymmetric parkinsonism, limb dystonia, myoclonus, saccadic apraxia, ideomotor apraxia, cortical sensory deficits, and alien limb phenomena are classic clinical clues for CBS. Like PSP, CBS also has varied phenotypic presentations apart from this classic phenotype. Though in the latest criteria for CBD by Armstrong et al. (30) four phenotypic presentations have been described, certain other presentations have been reported in the literature (Table 1). Cognitive presentation of CBD (CBD-Cog) mimicking bvFTD or AD is an increasingly recognized phenotype with apathy, executive dysfunction, language, and visuospatial problems (37). Asymmetric cortical atrophy predominantly affecting peri-rolandic region, posterior frontal, and parietal lobes (38) is the radiological hallmark of CBS, but the predominant area of atrophy varies with underlying pathology. CBS-CBD and CBS-PSP pathology: focal atrophy involving premotor cortex, posterior superior frontal lobe and supplementary motor area (SMA), CBS-TDP-43, and CBS-AD pathology: more widespread gray matter loss, CBS-TDP43 pathology: fronto-temporal involvement (particularly prefrontal cortex) and CBS-AD pathology: temporo-parietal involvement (particularly parietal cortex) (39). In 25–56% of cases, clinical diagnosis of CBS correlates with classic CBD pathology (CBD–CBS) (40). Astrocytic plaques, ballooned, or achromatic neurons and argyrophilic threads are pathological hallmarks of classic CBD pathology. However, CBS phenotype can be associated with various other pathological entities apart from classic CBD pathology like, AD pathology, PSP pathology, and FTLD-TDP43 pathology (40).

Because of the overlapping phenotypes, whether PSP and CBD are two different disorders or are part of a spectrum, is a matter of debate (41, 42). Phenotypically, PSP and CBD pathology both can present as speech/language (SL) dysfunction (agrammatic non-fluent aphasia/speech apraxia), frontal cognitive/behavioral presentation (F), Richardson's syndrome, and corticobasal syndrome (43). Interestingly, if a patient presents with VSGP or slowing of vertical saccade, axial or symmetric limb rigidity or akinesia, limb apraxia, and postural instability, the patient can be classified as PSP-CBS or CBD-PSP. To get rid of this conundrum, Movement Disorder Society (MDS) criteria (2017) for PSP have introduced the novel diagnostic category “probable 4R-tauopathy” for joint clinical recognition of the patients with PSP and CBD pathology and to facilitate the research on 4R-tau targeted therapeutic strategies (16, 44). Probable 4R-tauopathy includes “possible PSP with SL” and “possible PSP with CBS” apart from all “probable PSP.”

On top of this, vertical gaze palsy can be seen in a lot of other disorders apart from PSP-RS and asymmetric dystonic stiff limb presentation can be seen in other disorders besides CBS. Thus, clinicians should always be aware of these look-alikes (“mimics”) of PSP and CBS (45, 46) (Table 2).

**Table 2:** Mimics of PSP and CBS.

**Clinical entity**	**Look-alikes (Mimics)**	**Clinical clues**	**Radiological clues**
Progressive supranuclear palsy (PSP) (for classic PSP-RS with VSGP)	Niemann-Pick type C (NPC) (47, 48)	Splenomegaly, ataxia, dystonia, chorea, cognitive, and psychiatric symptoms, downgaze palsy, epilepsy, history of gelastic cataplexy, usual age of onset earlier than PSP (though can be late onset)	Frontal and cerebellar atrophy, white matter T2 hyperintensities in parieto-occipital periventricular regions
	Anti Ma2 related paraneoplastic syndrome (49, 50)	Hypothalamic- pituitary endocrine dysfunction, weight gain, sleep disorders (e.g., hypersomnia, narcolepsy, REM sleep behavioral disorders), rapid progression, history of testicular cancer	T2 FLAIR hyperintensities in mesial temporal, dorsal midbrain, medial thalamus and hypothalamus
	Anti IgLON5 related autoimmune disease (51, 52)	NREM and REM parasomnia, gait instability, cognitive impairment with or without chorea, autonomic dysfunction, bulbar dysfunction, sleep apnoea, and stridor	Mostly normal, may show brainstem, cerebellar and hippocampal atrophy, T2 FLAIR hyperintensities in hypothalamus and brainstem
	Anti LGI1 related autoimmune disease (53, 54)	Rapidly progressive dementia, facio-brachial dystonic seizure, hyponatremia, episodic bradycardia, humming	T2 FLAIR hyperintensities in bilateral hippocampus and medial temporal lobes
	Whipple's disease (55, 56)	Oculomasticatory myorhythmia, dementia, myoclonus, ataxia, history of frequent diarrhea, weight loss, arthralgia	T2 FLAIR hyperintensities and mildly contrast enhancing lesions in midbrain, mesial temporal lobe, hypothalamus and corticospinal tracts
	Frontotemporal lobar degeneration with MAPT gene mutation (FTLD-MAPT) (57, 58)	Family history of FTD-parkinsonism	Symmetric fronto-temporal atrophy
	Kufor-Rakeb disease (mutations in ATP13A2) (59, 60)	Juvenile onset, spasticity, facial-faucial-finger mini-myoclonus, upgaze palsy, oculogyric crisis, dementia, psychiatric features, levodopa responsive parkinsonism	Diffuse cerebral and cerebellar atrophy, increased iron accumulation can be seen in caudate and putamen in T2^*^/SWI MRI
	Mitochondrial disorders (Polymerase gamma/POLG1 gene mutations) (61)	Deafness, ataxia, epilepsy, migraine, neuropathy, positive family history	Cerebellar atrophy, T2 hyperintensities in cerebellar white matter, dorsal thalamus and inferior olivary nucleus
	Perry syndrome (mutations in DCTN1, TDP-43 proteinopathy) (62–64)	Unexpected weight loss, respiratory problem (hypoventilation), central sleep apnoea, apathy/depression, family history of parkinsonism or respiratory problems	Mostly normal, frontotemporal and midbrain atrophy can be seen
	Gaucher disease (Type 3) (mutations in GBA) (65)	Hepatosplenomegaly, horizontal > vertical gaze palsy and slow saccade, head thrusts, epilepsy, cognitive decline, usual age of onset earlier than PSP, ataxia, spasticity	Normal or mild diffuse cortical and midbrain atrophy
	Prion diseases like familial Creutzfeldt-Jakob disease (66, 67) or Gerstmann-Straussler-Scheinker disease (GSS) (mutations in PRNP) (68, 69)	Rapid progression, cognitive decline, myoclonus, ataxia	T2 FLAIR hyperintensity and DWI restriction in caudate, putamen and thalamus, cortical ribboning in DWI MRI, cerebellar atrophy
	Cerebral autosomal dominant arteriopathy with subcortical infarcts and leukoencephalopathy (CADASIL) (70, 71)	History of migraine, transient ischemic attacks/stroke, positive family history, cognitive decline (executive dysfunction), apathy or depression, subcortical white matter hyperintensities (mainly anterior temporal lobe, external capsule) in MRI brain	Periventricular white matter T2 hyperintensities and characteristic hyperintensities of anterior temporal lobe and external capsule
	Spastic paraplegia type 7 (SPG7) (72–74)	Spastic ataxia, optic neuropathy, bladder dysfunction (multisystem atrophy-cerebellar type /MSA-C mimicker)	Cerebellar atrophy
	Spinocerebellar ataxia type 2, type 3, type 17 (SCA2, SCA3, SCA17) (75–78)	Ataxia with parkinsonism, slow horizontal saccade (in SCA2), bulging eyes with upgaze palsy (in SCA3), autonomic dysfunction, cognitive decline and chorea (in SCA17), positive family history	Cerebellar atrophy
	Autosomal recessive parkinsonism due to Synaptojanin 1 (SYNJ1) gene mutation (45, 79)	Early onset parkinsonism, dystonia, with vertical supranuclear gaze palsy, history of seizure, cognitive decline	Diffuse cortical atrophy, thinning of quadrigeminal plate, hippocampal sclerosis
Corticobasal syndrome (CBS)	Frontotemporal lobar degeneration with Progranulin gene mutation (FTLD-PGRN) (57, 58)	Frontotemporal dementia associated with amyotrophic lateral sclerosis (FTD-ALS phenotype), family history of early onset dementia/ALS, language dysfunction, hallucination, prominent parietal signs like apraxia, dyscalculia, visuospatial impairment	Asymmetric fronto-temporal atrophy with temporo-parietal, parieto-occipital involvement
	Frontotemporal lobar degeneration with FUS, C9orf72 and TANK-binding kinase 1 (TBK1) gene mutation (80–84)	Frontotemporal dementia associated with amyotrophic lateral sclerosis (FTD-ALS phenotype), family history of dementia or ALS, history of hallucination, psychosis in C9orf72	Frontotemporal atrophy, additional caudate atrophy in FUS and cerebellar, thalamic atrophy in C9orf72
		Primary progressive aphasia (CBS-PNFA) in TBK1 mutation,	
	Familial and sporadic Creutzfeldt-Jakob disease (CJD) (85–88)	Rapid progression, cognitive decline, myoclonus, ataxia	T2 FLAIR hyperintensity and DWI restriction in caudate, putamen and thalamus, cortical ribboning in DWI MRI
	Vascular insults like multi infarct state (vascular CBS) (89–91)	History of transient ischemic attacks or stroke, dementia, history of dyslipidemia, ischemic heart disease, atrial fibrillation, peripheral vascular disease	MR evidence of multiple brain infarcts of different stages, stenosis of internal carotid arterial system in MR Angiography
	Antiphospholipid antibody syndrome (APLA) with or without cerebral infarction (92–94)	History of repeated pregnancy loss, deep vein thrombosis, chorea	Multiple T2 hyperintensities in subcortical white matter
	Presenilin 1 (PSEN1) mutation (95) (gene responsible for early onset Alzheimer's disease/EOAD)	Family history of dementia, earlier age of onset than classic CBS, seizure, cognitive impairment	Diffuse cortical atrophy including temporo-parietal lobe, subcortical and periventricular white matter T2 hyperintensities
	Amyloid precursor protein (APP) gene mutation (96, 97) (gene responsible for early onset Alzheimer's disease/EOAD)	Family history of dementia and/or parkinsonism, earlier age of onset than classic CBS, prominent cognitive impairment, with or without seizure	Medial temporal/Hippocampal atrophy
	Cerebrotendinous xanthomatosis (CTX) (98, 99) (mutation in CYP27A1)	Usual age of onset early than CBS, ataxia, tendon xanthoma, early cataract, cognitive decline, spasticity	Dentate and peri-dentate cerebellar white matter T2 hyperintensities
	Fahr's disease (Primary familial brain calcification/PFBC) (100)	Usual age of onset early than CBS, history of seizure, neuropsychiatric features including dementia, executive dysfunction and psychosis, positive family history	Evidence of bilateral brain calcification (basal ganglia, dentate, centrum semiovale) in CT or MRI
	Stiff limb syndrome (focal variant of stiff person syndrome) (101, 102)	Fluctuating stiffness (more with activity), tonic spasms provoked by tactile stimuli, anti GAD antibody positivity, ataxia, history of autoimmune diseases like type 1 diabetes, thyroiditis	Mostly normal, T2 FLAIR hyperintensities in medial temporal lobes can be seen
	Anti glycine receptor (anti-GlyR) antibody mediated (103, 104)	Rapid progression, hyperekplexia (excessive startle), progressive encephalomyelitis with rigidity and myoclonus (PERM), trigeminal/facial disturbance, ataxia	Mostly normal, subcortical and periventricular white matter T2 hyperintensities can be seen
	Diffuse Lewy body disease (DLB) (105, 106)	Fluctuating cognition, visual hallucination, delusion, neuroleptic sensitivity, autonomic dysfunction	Diffuse cortical atrophy (with relatively preserved medial temporal) in MRI and occipital hypoperfusion with “cingulate island sign” (preserved metabolism of the posterior cingulate) on SPECT/PET
	Adult-onset leukoencephalopathy with axonal spheroids and pigmented glia (ALSP) due to CSF1R gene mutation (107, 108)	Relative earlier onset than CBS, psychiatric symptoms with personality change, progressive cognitive decline, frontal executive dysfunction, pyramidal signs, history of seizure, rapid disease course	Dilation of the lateral ventricles, bilateral white matter T2 FLAIR hyperintensities with diffusion restriction, thinning of corpus callosum, abnormal signal intensities in corpus callosum and pyramidal tract, calcifications in the white matter

### Predominant Language Dysfunction Presentation

#### Progressive Nonfluent Aphasia (PNFA) or Agrammatic Variant of Primary Progressive Aphasia (PPA-G)

PNFA/PPA-G can clinically present with *apraxia of speech (AOS), agrammatism, or mixed*. AOS manifests as slow, labored, effortful, hesitant speech with inconsistent speech sound error and aprosody. “Groping after the target sound” is characteristic. Patients have difficulty to utter polysyllabic words and sequences of syllables (e.g., “puh-tuh-kuh”) (109). Phonemic speech sound errors are more common (110). Errors in grammar mainly affects syntax, function words, use of conjunction and verb (109). Clinically, PPA-G must be differentiated from the *semantic variant PPA-S* (single word comprehension and object knowledge is affected with intact repetition, commonly TDP43 pathology) and *logopenic variant PPA-L* (word finding difficulty with “tip-of-the-tongue” hesitation and impaired repetition, commonly AD pathology) (111). Orofacial apraxia is a common association with PPA-G. Signs of PSP or CBS may arise as the disease evolves (109). Predominately left peri-sylvian atrophy involving left posterior fronto-insular region (inferior frontal gyrus and insula) is seen in MRI brain (111). Around 30% of the cases are genetic and association with MAPT, PGRN and C9orf72 have been reported (112). PSP pathology is common in AOS variant with more dysarthric presentation and CBD pathology with more sentence comprehension deficit (113). Sometimes PiD pathology, TDP43-A pathology if there is associated ALS (nfvPPA-ALS) or AD pathology is also seen (112–114).

### Predominant Cognitive or Mixed Presentation

Three other primary tauopathies namely globular glial tauopathy (GGT), argyrophilic grain disease (AGD) and Pick's disease (PiD) present as cognitive or mixed (cognitive and movement disorder overlap). Clinical, radiological, and pathological features of these entities have been described in Table 3.

**Table 3:** Clinical, radiological, and pathological clues for GGT, AGD, and PiD.

**Disease entity**	**Clinical features**	**Radiology**	**Pathology**
GGT (28, 115, 116) (4R-tauopathy)	• Can clinically present with bvFTD (Type 1), PSP/CBS with MND/PLS spectrum (Type 2) and mixed (Type 3), based on topographic location of white matter deposits of tau immunoreactive globular glial inclusions (117) • Atypical PSP with marked upper motor neuron (UMN) signs (PSP-PLS phenotype) can be a clinical clue • Other phenotypes: PPA-G with chorea (118), PPA-S (119), Mill's hemiplegic variant of MND (120)	• Frontotemporal atrophy with T2 FLAIR hyperintensities in white matter involving cortical-white matter junctions, subcortical and periventricular areas, anterior commissure, posterior horn of lateral ventricles, cerebral peduncle, basis pontis (regions corresponding to traversing corticospinal fibers) (28, 120, 121)	Tau-immunoreactive globular inclusions in astrocytes (GAI) and oligodendrocytes (GOI) (117, 122) • *Type 1*: frontotemporal involvement • *Type 2*: motor cortex and/or corticospinal tract involvement • *Type 3*: frontotemporal, motor cortex and/or corticospinal tract involvement
AGD (123, 124)(4R-tauopathy)	• Limbic predominant 4R-tauopathy that commonly presents with very late onset (>75 year) slowly progressive mild cognitive impairment with prominent psychiatric symptoms (limbic involvement), disinhibited behaviors, change of appetite and eating disorders (hypothalamic involvement), late-onset schizophrenia and delusional disorders, late-onset bipolar disorder • AGD can be seen in very late onset CBD, PSP • CBD-Cog patients have found to have more AGD pathology than CBD-CBS (37) • Other phenotypes: Late onset Parkinson's disease with dementia, hallucination, delusion, mimicking Lewy body dementia (DLB) (125, 126), bvFTD presentation with diffuse cortical involvement (127) • Can rarely present with rapid cognitive decline, seizure, psychotic episodes, urinary incontinence in younger population (<75 year), mimicking prion disease (128)	• Medial temporal lobe atrophy • Midbrain atrophy if associated PSP pathology	• Argyrophilic grains spindle- or comma-shaped Gallyas positive, 4R tau in neuronal dendrites and axons • CBD, PSP, and AD pathology are commonly associated
PiD (129, 130) (3R-tauopathy)	• bvFTD (most common presentation), sometimes can present as PPA-S	• bvFTD: Frontal (predominantly medial frontal cortex and also involving dorsolateral and orbitofrontal regions) >temporal atrophy (131) • PPA-S: mainly left anterior temporal lobe, with involvement of inferior temporal gyrus, fusiform gyrus, anterior hippocampal region (113, 131)	Pick bodies, Pick cells, ramified astrocytes

### Secondary Tauopathies

#### Anti IgLON5 Disease

Anti IgLON5 mediated secondary tauopathy stands at a critical juncture of autoimmunity and neurodegeneration, where deposition of hyperphosphorylated tau (both 3R and 4R) occurs mainly in the hypothalamus, brainstem, and hippocampus. Initially, it was described as an antibody mediated sleep disorder (132, 133). Subsequently, many other phenotypes (chameleons) have emerged and most of the times they overlap (51, 134–137) (Table 4). MRI brain is mostly normal or may show cerebellar, brainstem atrophy (138). Recognition of these clinical phenotypes of anti IgLON5 are necessary because of its treatability with immunomodulators can prevent further neurodegeneration.

**Table 4:** Phenotypic presentations (chameleons) of IgLON5 disease.

Sleep disorders	NREM and REM parasomnias (commonly vocalization, simple or finalistic limb movements, RBD), sleep apnea and stridor, excessive daytime somnolence
Bulbar dysfunction	Dysphagia, dysarthria, laryngeal stridor, recurrent acute respiratory failure (mimicking ALS or myasthenia)
PSP phenotype	VSGP and gait instability (restriction in upgaze is more than downgaze in contrast to PSP)
MSA phenotype	Parasomnia, dysautonomia (urinary dysfunction, episodic profuse sweating), stridor, parkinsonism, ataxia
Acute or subacute encephalopathy	
Huntington's disease (HD) phenotype	Cognitive impairment with chorea
Orofacial dyskinesia	Facial myokymia and orolingual myorhythmia (mimicking Whipple's disease)
Motor neuron disease (MND) phenotype	Distal muscle atrophy, fasciculation
Stiff-person syndrome spectrum (SPS) phenotype	Peripheral nerve hyperexcitability with cramps, hyperekplexia, stiffness, myokymia, neuromyotonia
Cerebellar ataxia phenotype	Postural and intention tremor, titubation, gait, and limb ataxia
Cervical and truncal dystonia	

#### Chronic Traumatic Encephalopathy (CTE)

CTE is mainly a neurocognitive syndrome related to repeated traumatic brain injury (TBI) where both 3R- and 4R- tau deposition is seen (like AD). TBI likely ignites a vicious cycle of neuroinflammation and tau phosphorylation, deposition (139). Susceptibility depends on multiple factors like carrying ApoE4 allele, cognitive reserve, etc. It was initially described in boxers and named as “punch-drunk syndrome” or “dementia puglistica.” Subsequently, the disease got noticed among athletes like football players and war veterans. Gardner et al. (140) has classified the older ones as “*classic CTE*” (parkinsonism followed by cognitive symptoms) and the recent ones as “*modern CTE*” (behavioral symptoms affecting mood/affect followed by cognitive symptoms). From clinical perspective, Jordan et al. (141) have divided CTE into three phenotypes: (1) *Behavioral and psychiatric* (aggression, impulsivity, delusions, depression, suicidality) that can mimic bvFTD; (2) *Cognitive* (affecting attention, executive, memory, visuospatial domains) that can mimic FTD or AD; (3) *Motor* (parkinsonism, ataxia, dysarthria, spasticity). Chronic post-concussive syndrome (CPCS) comes as differential but its temporal relation with the acute concussive event and the presence of headache are the helpful differentiating features (139). Pathologically CTE differs from AD, though both are secondary tauopathy with mixed 3R- and 4R-tau deposition. Perivascular deposition of tau positive NFTs along the depth of cortical sulci is the pathological hallmark of CTE. TDP-43 inclusions are more common in CTE, while Aß amyloid deposition is more in AD (142). Additionally, tau filaments in CTE have a unique ß-helix region with a hydrophobic cavity, containing cofactors necessary for tau aggregation and propagation (143).

#### Alzheimer's Disease (AD)

AD is the most common cause of dementia worldwide. Pathologically, extracellular Aß amyloid plaques and intracellular tau (mixed 3R and 4R) positive neurofibrillary tangles are seen (144). Apart from classic amnestic presentation, non-amnestic phenotypes of AD are increasingly being recognized like, language variant (e.g., logopenic aphasia with word finding difficulty), visuospatial variant (posterior cortical atrophy/PCA with impaired spatial cognition), behavioral variant (executive dysfunction with impaired reasoning, problem solving), and mixed cognitive-motor presentation (atypical parkinsonism) (145, 146). In the latest National Institute on Aging and Alzheimer's Association (NIA-AA) Research Framework criteria (2018), AD has been re-defined based on the underlying pathology (amyloid pathology/A, tau pathology/T and neurodegeneration/N) that can be documented *in vivo* by biomarkers or by post-mortem examination (147). From a movement disorder perspective, many studies have reported extrapyramidal signs including parkinsonism in AD with widely varied prevalence (20–100%) (148). Parkinsonism in AD is mostly unresponsive to levodopa and the patients with AD-parkinsonism phenotype usually show relatively rapid progression, severe deficits on neuropsychological testing and high frequency of major depression and dysthymia (149). However, in these scenarios of cognitive-motor overlap, the clinicians must differentiate cortical “pseudo-parkinsonian” features like ideomotor apraxia, paratonic rigidity, and frontal/higher level gait disorders from true parkinsonian (nigrostriatal) features like bradykinesia, lead-pipe rigidity, and parkinsonian/middle-level gait disorders (148). Dementia with Lewy bodies/DLB (occurrence of dementia prior to or within a year of onset of motor symptoms, cognitive fluctuation, well-formed visual hallucination, neuroleptic sensitivity, and autonomic dysfunction), Parkinson's disease dementia/PDD (onset of dementia after 1 year of parkinsonian motor symptoms) and Creutzfeldt–Jakob disease/CJD (rapid progression, cerebellar ataxia, seizure, and chorea) can mimic AD-parkinsonism phenotype with dementia, rigidity, and myoclonus (149). Lastly, genes responsible for early onset AD (EOAD) like presenilin 1 (PSEN1) and amyloid precursor protein (APP) can give rise to atypical parkinsonism like corticobasal syndrome (CBS) (95, 96). Besides the CBS phenotype, dystonia in AD can also be drug-induced (e.g., rivastigmine, mirtazapine, neuroleptics) (150–152). Choline esterase inhibitors (ChEIs) can induce truncal dystonia in the form of Pisa syndrome (tonic lateral flexion of the trunk) in patients of AD (153–155). Apart from parkinsonism, cortical reflex myoclonus is common in advanced AD (in around 50%). Myoclonus can appear early in the disease course in early onset familial AD and in AD with faster progression (156). Small amplitude postural jerky tremor (minipolymyoclonus) has also been reported in AD (157).

### Geographically Isolated Tauopathies—Sociocultural and Environmental Impact

#### Guadeloupean Parkinsonism

High frequency of atypical parkinsonism with PSP like presentation is seen in French-Caribbean islands of Guadeloupe and Martinique (158). Two phenotypes have been described: (1) *Guadeloupean PSP-like syndrome (Gd-PSP)* with levodopa-resistant parkinsonism, early postural instability, and supranuclear gaze palsy (differs from classic PSP phenotype because of the high frequency of tremor, dysautonomia, and hallucination); (2) *Guadeloupean Parkinsonism-dementia complex (Gd-PDC)* with levodopa-resistant parkinsonism, subcortical dementia, and hallucination (9). Eating the fruits and infusions of the leaves of *Annona muricata* (soursop), containing Annonacin (toxic inhibitors of the mitochondrial respiratory chain complex I) has been proposed as a risk factor. Apart from supratentorial atrophy, 3rd ventricular dilatation (in both subgroups) and midbrain atrophy in Gd-PSP (like classic PSP), hypointense signals noted in T2 FLAIR, T2^*^ sequences over substantia nigra, red nucleus, globus pallidus, and putamen in both subgroups (an important radiological clue) (9). Pathologically, they can mimic PSP or may have some atypical features like absence of tufted astrocytes and more tau positive neurons than true NFT (159).

#### Western Pacific Amyotrophic Lateral Sclerosis and Parkinsonism-Dementia Complex (ALS/PDC)

Historically, epidemiologist Kurland and neurologist Mulder described the high incidence of atypical parkinsonism and familial ALS in Guam (southernmost of the Mariana islands) in native Chamorro tribe. Subsequently, Hirano et al. termed it as “Parkinsonism-dementia complex of Guam (PDC)” because of the common association of dementia (160). Three high-incidence foci have been described so far in the literature: (1) Guam, USA (“Lytico-bodig” disease in Chamorro tribe), (2) PapuaNew Guinea, Indonesia (Auyu and Jakai tribe) (161), and (3) Hohara and Kozagawa regions of Kii Peninsula, Honshu Island, Japan (“Muro” disease) (10). Though the clinical description varied in literature, most common presentation described was rapidly progressive, familial, symmetric akinetic-rigid parkinsonism (PSP or “*Bodig*” phenotype) along with distal muscle atrophy (ALS or “*Lytico*” phenotype), hyperreflexia, vertical gaze palsy, and dementia (160). While overall the incidence of ALS/PDC has decreased, in Kii peninsula it is still being reported because of the use of traditional medicines (10). Ophthalmomyiasis-like pigmentary retinopathy (“criss-crossed tracks of depigmentation” of the retinal pigment epithelium) has been reported in these patients of Kii peninsula (162). MRI brain shows rapidly progressive frontotemporal atrophy mainly in PDC subtype (163). Typical “Hummingbird sign” (164) has been reported too. Pathologically, ALS/PDC can be called a “multiple proteinopathy” because apart from widespread 3R- and 4R-tau positive neuronal and glial inclusions, NFTs throughout the gray and white matter, including in the lower motor neurons in the spinal cord, TDP-43 deposits and accumulation of alpha synuclein deposition as Lewy bodies and Lewy neurites were also noted in the amygdala, substantia nigra and locus coeruleus (165). Several etiological hypotheses exist in the literature. Ingestion of toxic chemicals in the flour from seed of cycad plants containing toxic β-methylamino-l-alanine (l-BMAA) and cycasin has been proposed in “cycad hypothesis.” Japanese folk medicine (Kampo) also contains *Sotetsu* seed (cycad) (10). Cyanobacteria (Blue-green algae) containing toxic BMAA reaches the cycad seeds via the roots and these native people cook bats who eat these cycad seeds (160). Interestingly, C9orf mutation has recently been reported in some of these people of Kii peninsula (166), but not in Guam (167).

#### Nodding Syndrome (NS)

Children in the East Africa, mostly the Acholi tribe in northern Uganda (“*lucluc*”), Wapogoro tribe of Tanzania (“*kifafa cha kusinzia*”) and South Sudan suffer from a deleterious syndrome initially presenting as stereotypical head dropping movements (triggered by food, cold weather) that gradually leads to cognitive impairment, malnutrition, impaired growth, seizures, epileptic encephalopathy, and parkinsonism in late stage (168, 169). Often the affected children die by accidental drowning and burns. Clinically, NS overlaps with sub-Saharan Nakalanga syndrome (NLS) with pituitary dwarfism (169). Several etiological hypotheses exist in the literature, autoimmune reaction toleiomodin-1 epitope of *Onchocerca volvulus* (nematode causing river blindness) has been mostly mentioned. Recently, widespread tau-immunoreactive NFTs and pre-tangles have been noted, mostly in the gyral crests of the frontal and temporal cortex, brainstem, substantia nigra, and locus coeruleus (11). MRI shows varying degree of cortical atrophy mainly involving fronto-temporal regions (170). Presence of tau pathology in NS is a pathological factor for the disease or just an effect of repeated seizure, is still to be determined.

#### Cluster of PSP in Northern France

Caparros-Lefebvre et al. (171) have reported a cluster of older onset (mean age 74 years) PSP cases (53% PSP-P, 33% PSP-RS) from suburban towns centered on Wattrelos and Leers, northern France. Etiopathogenesis has been linked to the environmental toxic exposure from the industrial dumping of phosphate and chromate ores in the territory.

Being familiar with these disease phenotypes are needed because many a times patients migrate and a so-called “geographically isolated” tauopathy can present to a clinician practicing far away.

### Novel Tauopathies—Are They?

Mulroy et al. (4) in their recent paper on “novel tauopathies,” have highlighted how pathological tau deposition is interestingly being noted in some other movement disorders like ADCY5 related dyskinesia, Beta-propeller protein associated neurodegeneration (BPAN), Benign hereditary chorea (BHC) Type 2, Huntington's disease (HD), Progressive ataxia, and palatal tremor (PAPT) and Spinocerebellar ataxia (SCA 11, 31). These are mostly isolated case reports and whether there is any pathological significance of tau in these disorders or tau is just an innocent bystander, is largely unknown. Presence of tau may be because of co-existing other known tauopathy, as a part of “mixed proteinopathy” or be just because of old age. Similarly, tau pathology is also seen in post encephalitic parkinsonism (172), Niemann-Pick type C disease (173) subacute sclerosing panencephalitis (SSPE) (174) and in prion disease like Gerstmann-Straussler-Scheinker disease (GSS) (175) although the clinico-pathological significance is still unknown.

## A Practical Clinical Approach to Tauopathies and Movement Disorders

**Figure 2:**
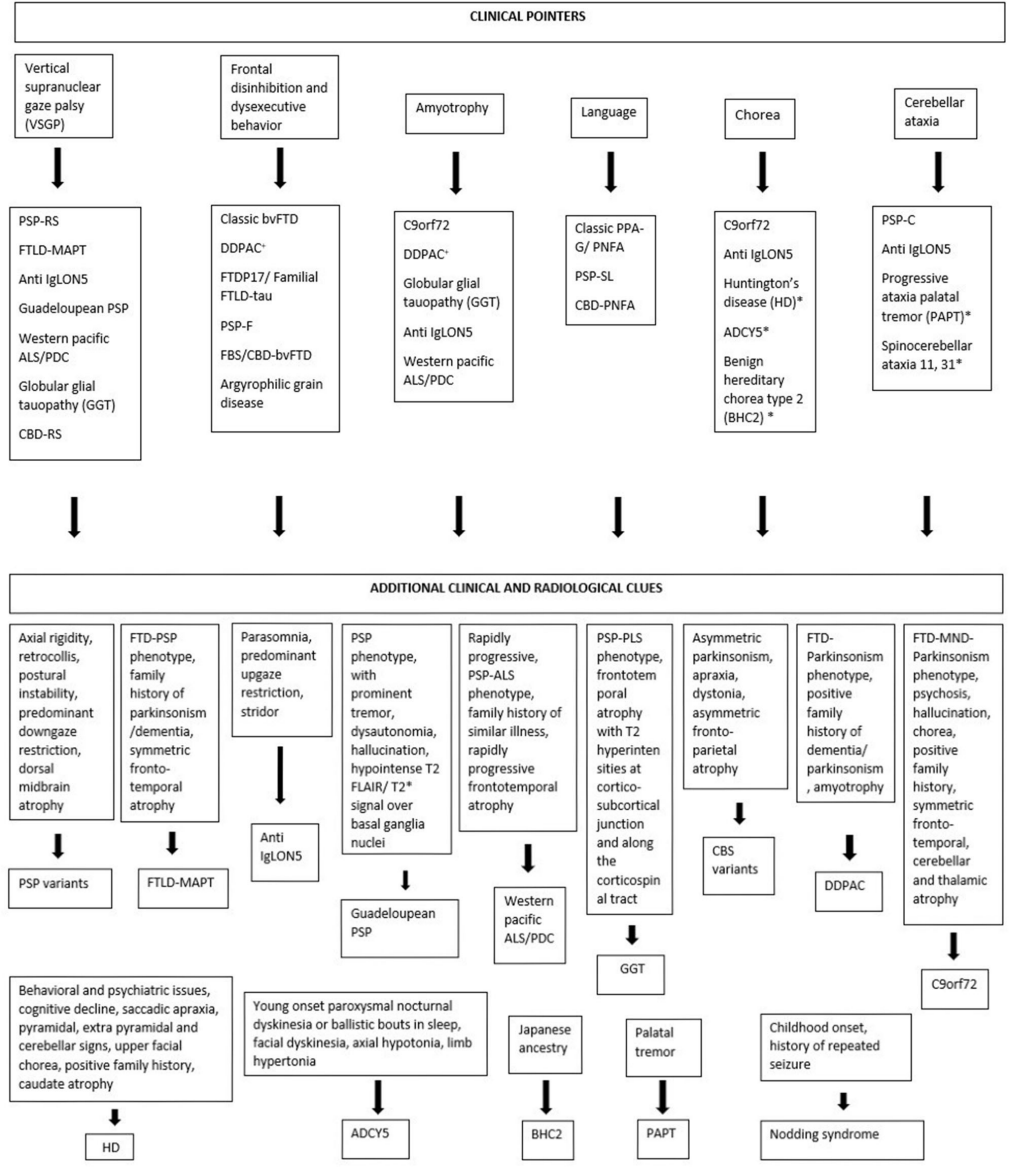
Clinical pointers for diagnosing tauopathies. *Novel tauopathies, +DDPAC, disinhibition–dementia–parkinsonism–amyotrophy complex related to MAPT mutation (intron 10 + 14).

Considering tauopathies from a movement disorder perspective, interlacing clinical phenotypes and pathological, genetic heterogeneity often make it difficult to diagnose them clinically. Some clinical pointers like VSGP, frontal disinhibited behavior, amyotrophy, prominent language involvement, chorea, and cerebellar ataxia can be helpful clues for the clinician on this regard (Figure 2).

## Familial FTD With Parkinsonism—A Phenotypic Overlap

The overlap of familial frontotemporal dementia and parkinsonism needs special attention because it is one of the commonest presenting phenotypes baffling the movement disorder specialists. Frontotemporal dementia and parkinsonism linked to chromosome 17 (FTDP-17) has recently been described as “familial FTLD-tau” because of the similarity of neuropathological features and disease progression between patients of familial FTLD-tau with MAPT mutations and sporadic FTLD-tau subtypes (PiD, PSP, CBD, and GGT) (176). Parkinsonism associated with familial FTD and MAPT mutation varies from mild to the aggressive form in severity and can occur early or late in this spectrum (57). Chromosome 17 carries another gene named progranulin (PGRN), that is also linked with the spectrum of frontotemporal dementia-parkinsonism, but with TAR DNA binding protein 43 (TDP43) inclusions instead of tau. Apart from these two common genetic associations (MAPT and PGRN), parkinsonism in familial FTD can also be liked with chromosome 9 open reading frame 72 (C9orf72) gene where overlap with motor neuron disease (FTD-MND) is commonly seen (177, 178) (**Table 6**). Four other less common genetic links reported in familial FTD with parkinsonism cases are—chromatin modifying protein 2B (CHMP2B), transactive response DNA-binding protein (TARDBP), valosin-containing protein (VCP), and fused-in-sarcoma (FUS) genes (179–181).

Phenotypically, MAPT and PGRN both can present with akinetic-rigid variant of parkinsonism. But MAPT commonly presents with PSP like phenotype with symmetric motor involvement while PGRN presents with corticobasal syndrome (CBS) like phenotype with asymmetric involvement and parietal lobe signs like apraxia, dyscalculia, visuoperceptual, and visuospatial dysfunction (57, 58, 182). Penetrance is 100% in MAPT, while it is age dependent in PGRN and reaches about 90% at the age of 70 (58). So, if there is no family history, MAPT is unlikely but PGRN can still be a possibility. Progression of disease is relatively faster in PGRN (131) and hallucinations (183) are more common. Radiologically, symmetric fronto-temporal atrophy is seen in MAPT involving anteromedial temporal lobe and orbitofrontal region (184) while caudate atrophy (185) (also common in FUS) (186) can also be seen. However, PGRN commonly presents with asymmetric fronto-temporal atrophy and more prominent posterior atrophy involving temporo-parietal, parieto-occipital regions (58, 182). White matter hyper-intensities are more frequent in PGRN (187). Additional cerebellar and thalamic atrophy can be seen in C9orf72 along with symmetric frontotemporal atrophy (188, 189) (Table 5).

**Table 5:** Clinical and radiological clues for familial FTD with Parkinsonism.

**Clinical clues**	**Radiological clues**	**Targeted gene**	**Suspected pathology**
Early onset (3rd or 4th decade), PSP phenotype, vertical supranuclear gaze palsy	Symmetric fronto-temporal atrophy	MAPT	Tau
Late onset (5th or 6th decade), CBS phenotype, FTD-MND overlap, language involvement, apraxia, dyscalculia, visuospatial impairment, episodic memory involvement, hallucination	Asymmetric fronto-temporal atrophy, more posterior involvement (temporo-parietal, parieto-occipital), significant white matter hyperintensities	PGRN	TDP43
FTD-MND overlap, early cognitive, and/or behavioral symptoms, psychosis, hallucination, chorea (Huntington's disease phenocopy), positive family history of MND or FTD	Symmetric fronto-temporal and cerebellar atrophy	C9orf72	TDP43, Ubiquitin

In addition to this, specific mutations in the MAPT gene can present with subtle phenotypic differences (57) (Table 6). On the other hand, Forrest et al. have noted pathological variability with specific mutations in MAPT like PSP pathology in S305S, CBD pathology in S305S, IVS10+16 and R406W, PiD pathology in K257T and GGT pathology in P301L, IVS10+16 mutation (176).

**Table 6:** Clinical phenotypes associated with MAPT gene mutations.

**Clinical Phenotype**	**MAPT gene mutation**
Early prominent personality change with disinhibition–dementia–parkinsonism–amyotrophy complex (DDPAC)	intron 10 + 14 (190, 191)
Early onset aggressive parkinsonism	N279K, P301S, intron 10 + 16,G389R, intron 10 + 13 (57)
CBS phenotype	N410H, P301S (192, 193) G389R, C291R (96)
Rest tremor (uncommon in FTD-parkinsonism)	K317M, G389R, Q336H (194, 195)

## Conclusion

Tauopathy is a complex clinico-pathological hub encompassing multiple facets of movement disorders, dementia, and motor neuron disease. Topographic localization of tau accumulation shapes the clinical phenotype with varied combination of these domains. They can present with diverse overlapping phenotypes and their presentation can be mimicked by a lot of other diseases. Recognizing these “chameleons” and “mimics” are necessary from clinical and therapeutic standpoints. Emerging secondary tauopathies and geographically isolated tauopathies, many a time having relation with secondary environmental factors, are continuously invoking more and more research on the pathogenesis of tauopathy. Obviously, we can't stamp a disorder as “tauopathy” by mere presence of tau pathology in the brain. But is there a reliable clinical criterion for tagging a disorder as “tauopathy” ? MDS-PSP criteria has introduced the diagnostic category of “probable 4R-tauopathy” for ante mortem diagnosis of patients with PSP or CBD pathology (16). However, the spectrum of tauopathy is expanding far beyond these two pathological subtypes and tau deposition is being seen in different disease entities. Confirming the role of tau as a pathogenic factor for these disorders is the unmet need of the hour. The crosstalk between autoimmunity and neurodegeneration or neuroinflammation and tau aggregation also demands further research on this regard (196).

Traditionally, tauopathy has been depicted in the literature either as a pathological construct or from a cognitive perspective, while the evolving movement disorder domain of tauopathy is often neglected. Intertwining of the clinical, radiological, genetic, and pathological domains of movement disorder makes the spectrum intriguing but creates diagnostic confusion. Subtle clinical and radiological clues are the keys here to navigate through this conundrum. They are not only helpful for targeted genetic testing and predicting the pathology before autopsy, but also can open the door for utilizing newer biomarkers like ligand gated imaging or CSF biochemistry more efficiently and encourage further research on protein based therapeutic strategies.

## Author Contributions

JG: organization and execution of the research project and writing of the first draft of the manuscript. MJ: conception of the research project and review and critique of the manuscript. Both authors contributed to the article and approved the submitted version.

## Conflict of Interest

The authors declare that the research was conducted in the absence of any commercial or financial relationships that could be construed as a potential conflict of interest.
